# Optimal Collaborative Management of Patients With Esophagogastric Cancers

**DOI:** 10.6004/jadpro.2017.8.3.4

**Published:** 2017-04-01

**Authors:** David Ilson, Steve Malangone

**Affiliations:** 1 Memorial Sloan Kettering Cancer Center, New York, New York;; 2 University of Arizona Cancer Center, Tucson, Arizona

## Abstract

The addition of radiation to preoperative chemotherapy and newer therapeutic combinations add to treatment options for these difficult-to-treat cancers.

Esophageal and gastric cancers account for 2.6% of all malignancies. Survival has improved over the past 3 decades, but it still remains only about 30% at 5 years for those with this type of cancer.

"Clearly, treatments that go beyond surgery alone are indicated," said David Ilson, MD, PhD, of Memorial Sloan Kettering Cancer Center, New York, who discussed this malignancy at JADPRO Live, along with Steve Malangone, MSN, FNP-C, AOCNP®, of the University of Arizona Cancer Center, Tucson.

## STAGING

Staging of esophageal and gastric cancers begins with esophagogastroduodenoscopy and biopsy. CT scanning of the chest, abdomen, and pelvis is important for identifying metastases. Endoscopic ultrasound gives an accurate tumor and nodal stage, which helps to guide treatment.

"The vast majority of patients we see are T3 or node-positive, and for these patients, we do laparoscopy and PET scan," Dr. Ilson said. According to a Surveillance, Epidemiology, and End Results (SEER) database, patients with gastric cancer who have T3/T4 tumors and those with disease in the lymph nodes have a 3-year survival of less than 40% (McGhan, Pockaj, Gray, Bagaria, & Wasif, 2012).

Laparoscopy yields positive cytology or peritoneal liver findings in one-quarter or more of patients, which upstages the patient and alters the treatment plan. Positron-emission tomography scan identifies occult metastases in 15%. When patients have metastatic disease at baseline, rather than undergo neoadjuvant chemotherapy and surgery, treatment shifts to chemotherapy alone.

Dr. Ilson emphasized that for patients with "obvious" metastatic involvement, CT imaging is sufficient; they warrant a more expensive PET scan only if the disease is not visualized on CT.

"For early T1 tumors, we consider endoscopic mucosal resection. For T1b and T2 disease, we do surgery. And we use neoadjuvant therapy or combined-modality treatment for T3 or node-positive disease," Dr. Ilson said.

## NEOADJUVANT AND ADJUVANT THERAPIES

For the treatment of gastric cancer, there are three accepted approaches, all of which include chemotherapy and resection:

- Surgery followed by postoperative (adjuvant) chemotherapy with fluorouracil (5-FU) and radiotherapy, primarily for patients in whom resection was less than level D1 (i.e., a suboptimal number of nodes were resected);
- Preoperative and postoperative chemotherapy with epirubicin, cisplatin, and 5-FU (ECF), for patients with D1–2 resection;
- Postoperative chemotherapy with capecit-abine and oxaliplatin (XELOX) for patients with D2 resection.

"The most common approach for gastric cancer in the United States is either pre- and postoperative chemotherapy, or surgery followed by a combination of chemotherapy and radiation," stated Dr. Ilson. "I think if you do upfront surgery with a D2 resection in a patient with stage 3 disease, the treatment is capecitabine and oxaliplatin. If you do upfront surgery and only eight to nine nodes were resected, consider postoperative 5-FU and radiation," he advised.

For esophageal and gastroesophageal junction (GEJ) cancers, the approach is a little different, as chemotherapy alone is not sufficient and radiation therapy has an established role. "For this cancer, I draw a firmer line. Chemotherapy alone is not enough," Dr. Ilson said.

Studies have yielded mixed results with preoperative chemotherapy in esophageal and GEJ cancers. Some, but not all, studies showed a benefit, and in essentially all studies, the rate of R0 resection (i.e., negative margins) was low, 60% to 70%, indicating a high risk for recurrence and death. This was true even for the more recent study that incorporated endoscopic ultrasound and PET staging (Alderson et al., 2015). "You are writing off 30% to 40% of your patients by giving them preoperative chemotherapy alone," he noted.

The addition of radiation to preoperative chemotherapy proved successful in the 2012 Dutch CROSS trial, and thus it became the global standard of care in esophageal and GEJ cancers (van Hagen et al., 2012). The "modern, easy-to-give regimen" involves five cycles of weekly bolus paclitaxel and carboplatin, with concurrent radiation, followed by surgery. This regimen led to a 13% improvement in 5-year overall survival (Shapiro et al., 2015). "The key was that the R0 resection rate increased to 92% with concurrent chemoradiation," he said. "Chemoradiation gives patients a better chance at curative resection."

## NEWER THERAPEUTIC COMBINATIONS

Results of the CROSS trial elevated carboplatin/paclitaxel plus radiotherapy to the standard of care, but alternative regimens have also been found beneficial in other studies, including chemoradiotherapy with irinotecan/cisplatin or paclitaxel/cisplatin in ECOG 1202 (Kleinberg et al., 2007), chemoradiotherapy with oxaliplatin/5-FU in SWOG S0-356 (Leichman et al., 2011), and chemoradiotherapy with 5-FU/cisplatin or FOLFOX (folinic acid, 5-FU, oxaliplatin) in a French trial (Conroy et al., 2014; but Dr. Ilson considers 5-FU plus cisplatin "excessively toxic").

Although the previously mentioned regimens have improved overall survival by 10% to 15%, more than 50% of patients still succumb to their disease. Additional cycles of chemotherapy are not beneficial, but maintenance therapy with targeted agents or immunotherapies may prove helpful in the future, especially in high-risk patients with residual tumor, and should be studied in well-designed, controlled clinical trials.

## PREOPERATIVE ASSESSMENT OF RESPONSE WITH PET SCAN

The benefit of preoperative chemotherapy appears limited to patients who demonstrate responses on PET scanning, based on a landmark study in which PET responders had a 3-year overall survival rate of 70%, compared with 35% for nonresponders (Ott et al., 2006). The German MUNICON-1 trial took the concept a step further, using PET to guide decision-making (Lordick et al., 2007). Nonresponders discontinued treatment, whereas responders continued on therapy. These early nonresponders were twice as likely to die of their disease; continuation of chemotherapy did not benefit them, and discontinuation of chemotherapy did not harm them, Dr. Ilson noted.

"We learned that PET response is an important biomarker during preoperative chemotherapy. Without a response, you may as well stop the chemotherapy and send the patient right to surgery or change the chemotherapy regimen," he added.

CALGB 80803, which is evaluating the benefit of switching treatment for nonresponders, has been completed but not reported. "The study met its primary endpoint, suggesting that early PET scanning to direct preoperative therapy may improve outcomes in nonresponding patients," revealed Dr. Ilson.

## A TYPICAL PATIENT WITH LOCALLY ADVANCED DISEASE

"Gastric and GEJ cancers are complicated diseases to treat," said Mr. Malangone, who described management strategies for a typical patient.

The patient presented with 6 months of worsening dysphagia, a 20-pound unintended weight loss, low-grade anemia, reduced albumin, and normal electrolytes and renal function. Esophagogastroduodenoscopy revealed a locally advanced GEJ cancer but no metastases. Endoscopic ultrasound revealed an enlarged lymph node, confirmed as adenocarcinoma by fine-needle aspiration.

The patient was referred to a nutritionist, radiation oncologist, and thoracic surgeon, and the case was discussed in the multidisciplinary tumor conference. The surgeon placed a jejunostomy tube, which is important when there is a risk for obstruction.

"You need to take dysphagia into consideration. Obstruction can happen, so think ahead. With locally advanced disease, think about supportive nutrition, and consider placing an enterostomy tube," Mr. Malangone said. "In patients with metastatic disease, I have found that a big quality-of-life issue is not being able to eat, and it’s important to palliate. Options include radiation, stenting, and dilation."

The consensus recommendation for this patient was neoadjuvant chemotherapy with weekly carboplatin and paclitaxel, as per the CROSS study (van Hagen et al., 2012), and concurrent radiotherapy, followed by surgery. He tolerated the therapy well overall, with only grade 1 fatigue, nausea, and neutropenia, which did not require treatment delays, dose reductions, or growth factor support.

However, by week 4, the patient reported worsening of dysphagia and esophagitis, with substernal pain, and demonstrated another 10-pound weight loss. He was dehydrated and malnourished, and his albumin had dropped. He was treated with intravenous hydration in the clinic and empirically with famotidine at 20 mg twice daily and viscous lidocaine as needed, with good relief of symptoms. Had these measures not been effective, the patient would have been referred for repeat esophagogastroduodenoscopy. At this time, the nutritionist instituted feedings with an enteral tube.

"The patient went on to complete his therapy without interruption. A follow-up PET/CT scan 6 weeks after radiation showed a near complete response. He underwent an esophagectomy 8 weeks after completion of neoadjuvant therapy," Mr. Malangone reported. The final pathology report showed adenocarcinoma, minimal focal residual disease, negative margins, and no metastatic nodal involvement.

"He was downstaged from T4N1 disease to T1N0," revealed Mr. Malangone. "He has a fairly good prognosis now, compared with the start of therapy, and is being followed in our survivorship clinic." This assessment is based on the large retrospective study by Chirieac et al. (2005), showing posttherapy pathology stage as the most predictive prognostic indicator in patients treated for gastroesophageal junction adenocarcinoma with trimodality therapy.

## TREATMENT APPROACHES TO METASTATIC DISEASE

Approximately 40% of patients have metastatic disease at diagnosis, and about 50% of those with earlier disease, will develop metastatic disease. Ultimately, most patients will receive palliative chemotherapy. Chemotherapy modestly improves survival, vs. best supportive care, with no real differences among the regimens (Table 1). Response rates are approximately 40%, median overall survival ranges from 9 to 10 months, and time to disease progression is 6 months, Dr. Ilson noted.

**Table 1:**
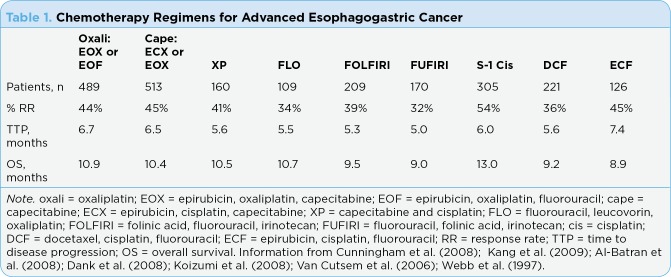
Chemotherapy Regimens for Advanced Esophagogastric Cancer

In selecting patients for chemotherapy, consider their age, functional status, and comorbidities. Doublets are preferred over single agents, except for elderly patients with a poor functional status, whereas triplets add more toxicity than benefit over doublets. "It doesn’t really matter which you use," admitted Dr. Ilson, "but I’m not a fan of DCF [docetaxel, cisplatin, 5-FU) or ECF, as they are more toxic."

In TAX 325, the addition of docetaxel to cisplatin/5-FU reduced disease progression by 32%, extended the time to disease progression by 2 months, and added 6 months to median overall survival, but nearly half the patients discontinued DCF because of toxicity (Van Cutsem et al., 2006). The modified lower-dose schedule can still be difficult to tolerate, as shown in a 2015 study in which one of five patients was hospitalized for toxicity (Shah et al., 2015).

"Even the modified schedule is a spicy cocktail that I don’t think most patients should be exposed to," he commented. The addition of docetaxel to oxaliplatin and leucovorin similarly increases toxicity.

Dr. Ilson also prefers infusional 5-FU over capecitabine, as cumulative skin toxicity is less with 5-FU. He advised using oxaliplatin over cisplatin and cautioned that epirubicin should not be used at all, due to excess toxicity. For second-line chemotherapy, standard options include a taxane or irinotecan.

## BEYOND CHEMOTHERAPY: RECENT SUCCESSES

Since conventional chemotherapy has limited efficacy, the focus of drug development is targeted treatments and immunotherapies. Targeted agents attempt to block specific tumor-growth pathways and include monoclonal antibodies, tyrosine kinase inhibitors, soluble receptors to growth factors, and downstream pathway inhibitors.

Among these targeted agents, the most success has been achieved with the anti-HER2 agent trastuzumab (Herceptin). In the phase III TOGA trial, trastuzumab added to chemotherapy significantly improved overall survival in metastatic patients (*p* = .0046); patients with high expression of HER2 derived an additional 4.2 months (Bang et al., 2010). To the contrary, the anti-HER2 agent lapatinib (Tykerb) failed to produce a benefit over chemotherapy alone in the LOGIC trial (Hecht et al., 2013). In the second-line setting, ado-trastuzumab emtansine (Kadcyla; formerly known as TDM-1) proved no better than paclitaxel in the GATSBY trial (ADC Review, 2015).

Other HER2-directed therapies are being evaluated. In the first-line setting, the JACOB trial is evaluating dual HER2 targeting with trastuzumab plus pertuzumab (Perjeta), and HELOISE is evaluating trastuzumab plus capecitabine/cisplatin. In early-stage disease, RTOG 1010 is evaluating preoperative chemoradiation therapy with or without trastuzumab, along with maintenance trastuzumab.

Except for HER2, biomarkers are lacking in gastric cancer. Recent trials of agents targeting the epidermal growth factor receptor (EGFR) and vascular endothelial growth factor receptor (VEGFR) have largely failed in unselected patient populations.

"But we do have positive results for ramucirumab (Cyramza) in the second-line treatment of gastric cancer," he added. In the RAINBOW trial, ramucirumab, which targets VEGFR, improved overall survival by 2.3 months (Wilke et al., 2014). "The almost 10-month median survival in the second line set a new benchmark and makes paclitaxel plus ramucirumab the standard of care," said Dr. Ilson. In the first-line setting, RAINFALL is evaluating ramucirumab with capecitabine/5-FU/cisplatin.

## GENOMIC PROFILING: LITTLE TO SHOW, SO FAR

The hope is that genomic profiling of gastric cancer will identify more targets to attack, but little useful information has been gained so far. While interest by patients is high, and cancer centers promise "magic cures," for at least 95% of patients, genomic profiling "yields no useful information," Dr. Ilson emphasized.

The Cancer Genome Atlas is using multiple types of genomic and proteomic platforms to profile esophagogastric tumors. Among 149 esophageal adenocarcinomas, 26 significant genes with mutations or genomic loss have been identified (Dulak et al., 2013). Interestingly, genes that are commonly mutated in other tumors, such as *KRAS*, *BRAF*, *ERBB2*, and *EGFR*, were not among them. Gene amplification, on the other hand, occurred in 37% of tumors and included *EGFR*, *ERBB2*, *MET*, *FGFR1-2*, and *KRAS*—potentially targetable factors. However, inhibition of *EGFR* and of *MET* has universally failed to improve outcomes, and results are mixed for the targeting of *ERBB2*.

What has emerged from The Cancer Genome Atlas that may prove clinically important are four distinct genomic subsets of upper gastrointestinal cancers. One of these subtypes demonstrates factors that are indicative of immune responsiveness. The fact that in gastric cancer, expression of programmed cell death ligands 1 and 2 (PD-L1/2) is elevated and there is a "high mutational load" could bode well for anti–PD-1/PD-L1 antibodies (Muro et al., 2016). With activity shown for the anti–PD-1 drugs pembrolizumab (Keytruda) and nivolumab (Opdivo), clinical trials of these and other immunotherapies are underway (Table 2).

**Table 2:**
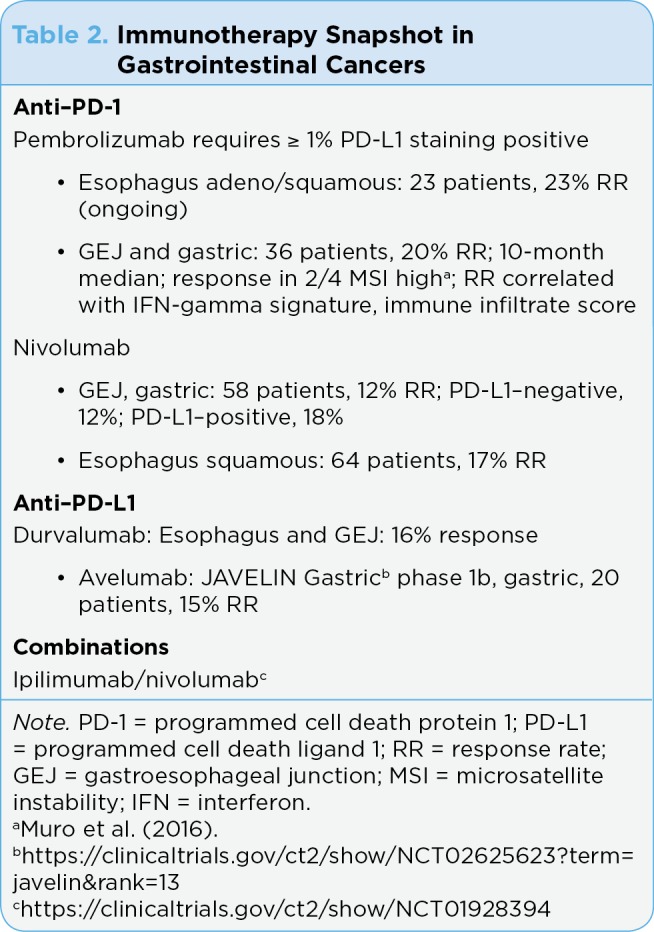
Immunotherapy Snapshot in Gastrointestinal Cancers
